# Transcriptional Characterization of a Widely-Used Grapevine Rootstock Genotype under Different Iron-Limited Conditions

**DOI:** 10.3389/fpls.2016.01994

**Published:** 2017-01-05

**Authors:** Alessandro Vannozzi, Silvia Donnini, Gianpiero Vigani, Massimiliano Corso, Giorgio Valle, Nicola Vitulo, Claudio Bonghi, Graziano Zocchi, Margherita Lucchin

**Affiliations:** ^1^Dipartimento di Agronomia Animali Alimenti Risorse Naturali e Ambiente, Università di PadovaLegnaro, Italy; ^2^Centro Interdipartimentale per la Ricerca in Viticoltura ed EnologiaConegliano, Italy; ^3^Dipartimento di Scienze Agrarie e Ambientali, Università di MilanoMilano, Italy; ^4^Centro di Ricerca Interdipartimentale per le Biotecnologie InnovativePadova, Italy

**Keywords:** *Vitis*, ferrome, mRNA-Seq, strategy I, micronutrients

## Abstract

Iron chlorosis is a serious deficiency that affects orchards and vineyards reducing quality and yield production. Chlorotic plants show abnormal photosynthesis and yellowing shoots. In grapevine iron uptake and homeostasis are most likely controlled by a mechanism known as “Strategy I,” characteristic of non-graminaceous plants and based on a system of soil acidification, iron reduction and transporter-mediated uptake. Nowadays, grafting of varieties of economic interest on tolerant rootstocks is widely used practice against many biotic and abiotic stresses. Nevertheless, many interspecific rootstocks, and in particular those obtained by crossing exclusively *non-vinifera* genotypes, can show limited nutrient uptake and transport, in particular for what concerns iron. In the present study, 101.14, a commonly used rootstock characterized by susceptibility to iron chlorosis was subjected to both Fe-absence and Fe-limiting conditions. Grapevine plantlets were grown in control, Fe-deprived, and bicarbonate-supplemented hydroponic solutions. Whole transcriptome analyses, via mRNA-Seq, were performed on root apices of stressed and unstressed plants. Analysis of differentially expressed genes (DEGs) confirmed that Strategy I is the mechanism responsible for iron uptake in grapevine, since many orthologs genes to the *Arabidopsis* “ferrome” were differentially regulated in stressed plant. Molecular differences in the plant responses to Fe absence and presence of bicarbonate were also identified indicating the two treatments are able to induce response-mechanisms only partially overlapping. Finally, we measured the expression of a subset of genes differentially expressed in 101.14 (such as *IRT1, FERRITIN1, bHLH38*/*39*) or known to be fundamental in the “strategy I” mechanism (*AHA2* and *FRO2*) also in a tolerant rootstock (M1) finding important differences which could be responsible for the different degrees of tolerance observed.

## Introduction

Nowadays, much of the European viticulture is based primarily on grafting, where the scion is represented by a *Vitis vinifera* cultivar and the rootstock is either a North American *Vitis* species or an interspecific *Vitis* hybrid carrying the resistance to phylloxera (Weaver, 1976). Although, at first, the replacement of the entire root system of “*vinifera*” varieties with “*non-vinifera*” or “American” species was seen as a sort of contamination of the purity and quality of wine, subsequently, the use of grapevine rootstocks spread also over those countries where viticulture constituted a young and new challenge and where phylloxera did not represent a menace yet (Arrigo and Arnold, 2007; Gregory et al., 2013). The explanation of this trend relies on the fact that rootstocks were found not only to confer resistance to diseases, but also to imply a larger range of advantages, controlling numerous physiological processes at the level of scion such as biomass accumulation (Gregory et al., 2013), fruit quality (Walker et al., 2002, 2004), grape berry development and ripening (Corso et al., 2016), and the ability to respond to many biotic and abiotic stresses (Marguerit et al., 2012; Meggio et al., 2014) including salinity (Fisarakis et al., 2001) and drought (Gambetta et al., 2012; Marguerit et al., 2012; Corso et al., 2015). Despite this, since their first introduction in the agronomical practice, one of the problems winegrowers had to cope with was the adaptation of these American species to European soils. In fact, sometimes interspecific rootstocks, and in particular those ones obtained by crossing exclusively *non-vinifera* species such as the widely-used 101.14 genotype (*V. riparia* × *V. rupestris*), can show several problems related to the nutrient uptake (Bavaresco et al., 1993) in particular for what concerns iron (Fe). Although this element is quite abundant in the earth's crust as well as in cultivated soils, its acquisition by plants is often impaired since it is insoluble in well-oxygenated and alkaline environments (such as calcareous soils) being mainly accumulated as oxi-hydroxide compounds (Tagliavini and Rombolà, 2001). Among the essential micronutrients in plants, Fe is required in the greatest abundance. Because its capability to change redox state and being the key component of both hemoproteins and iron sulfur proteins (including cytochromes, catalases, peroxidases, and ferredoxin) it plays a pivotal role in many important cellular processes such as photosynthesis, respiration, chlorophyll biosynthesis, nitrogen, and sulfur assimilation, hormone biosynthesis, etc. (Hänsch and Mendel, 2009; Kobayashi and Nishizawa, 2012; Balk and Schaedler, 2014; Briat et al., 2015). To give an idea of the global importance of Fe fine tune regulation in all these fundamental biologic processes, on the one hand it was demonstrated to be a limiting factor for biomass and seed yield in different crops including tomato (*Solanum lycopersicum*; Jin et al., 2009), spinach (*Spinacea oleracea*; Jin et al., 2013), and rice (*Oryza sativa*; Takahashi et al., 2001). On the other hand, its excess is toxic to plant cells due to Fe ability to change its oxidation status that leads to ROS production (Thomine and Lanquar, 2011).

In order to counteract the low availability of Fe in the soil, during their evolution plants have adopted two main uptake mechanisms, proposed for the first time by Römheld and Marschner (1986), and known as Strategy I in non-graminaceous plants and Strategy II in graminaceous plants. Since the end of the 1990s, many different genes involved in these strategies have been identified and almost perfectly fit the model scheme proposed by Römheld and Marschner. The Strategy I consists of three main steps: (a) the reduction of Fe(III)–Fe(II) by a ferric reductase oxidase enzyme (FRO2) located in the apoplast (Robinson et al., 1999); (b) the import of the resulting Fe(II) ions through the plasmalemma by the activity of a divalent iron-regulated transporter (IRT1; Eide et al., 1996; Vert et al., 2002) belonging to the ZIP multigenic family (Guerinot, 2000) and (c) the acidification of local rhizosphere mediated by plasma membrane H^+^-ATPase (AHA2/7) proton extrusion (Dell'Orto et al., 2002; Santi and Schmidt, 2009). Orthologs of Arabidopsis *AHA2, FRO2*, and *IRT1* genes have been identified and studied in various plant species including bean (*Pisum sativum*; Waters et al., 2002), tomato (*Solanum lycopersicon*; Zamboni et al., 2012), peanut (*Arachis hypogea*; Xiong et al., 2014), cucumber (*Cucumis sativus*; Santi et al., 2005). Although the key steps of Strategy I iron uptake have been exhaustively described, many other processes seem to be involved in the mechanisms of Fe uptake such as the production and subsequent excretion of iron-chelators. The type of chelators released by the roots appears to be species specific, for example flavins for tobacco (*Nicotiana tabacum*) and *Medicago trucantula* (Vorwieger et al., 2007; Rodríguez-Celma et al., 2013), and phenolic compounds for Arabidopsis and red clover (*Trifolium pratense*; Jin et al., 2007; Fourcroy et al., 2014; Schmidt et al., 2014). Plant responses to Fe deficiency have been recently analyzed on the basis of large-scale changes not only in transcriptome (Thimm et al., 2001; O'Rourke et al., 2007a,b, 2009; Buckhout et al., 2009; Forner-Giner et al., 2010; Yang et al., 2010), but also in proteome (Brumbarova et al., 2008; Li et al., 2008; Donnini et al., 2010; Lan et al., 2011) and metabolome (Rellán-Álvarez et al., 2009). Recently, a set of 92 transcripts that robustly reflect the transcriptional response of *Arabidopsis* to Fe deficiency has been described as the “*ferrome*” by Schmidt and Buckhout (2011) and consists of a list of genes considered to be involved in the basic response to iron deficiency.

Focusing on grapevine, the only information available on the plant response to Fe deficiency derives from several physiological and biochemical studies (Brancadoro et al., 1995; Nikolic et al., 2000; Bertamini et al., 2002; Piagnani et al., 2003; Bavaresco et al., 2006; Russo et al., 2010), most of which performed on *Vinifera* varieties, not considering that in the majority of cases, roots, which are the first organ perceiving the stress and actively responding to it, belong to American or interspecific hybrids.

In the present study we performed a physiological and genome-wide transcriptional characterization of 101.14 (*V. riparia* × *V. rupestris*), a commonly used grapevine rootstock susceptible to iron chlorosis, in response to Fe deficiency. Not only we analyzed the transcriptome effect of iron deprivation itself, but we also compared it to a condition of low iron availability determined by high concentration of bicarbonate, a phenomenon quite common in nature, which better mimics what happens in the vineyard. Our approach led to the identification of a set of genes differentially expressed (DEG) in response to these nutritional stresses. Many DEGs were orthologs of the so-called *Arabidopsis* ferrome, confirming the grapevine roots behave similarly to *Arabidopsis* under iron shortage, undertaking a Strategy I mechanism of iron homeostasis. Finally, we compared the expression of a subset of genes found to be differentially regulated in 101.14 (such as *IRT1, FERRITIN1, bHLH38*/*39*) or known to be fundamental in the “strategy I” mechanism (*AHA2* and FRO2) both in 101.14 and in a tolerant experimental rootstock (M1), finding important differences which could be responsible for the different degrees of resistance observed.

## Materials and methods

### Plant material and stress treatments

Plants of 101.14 (*Vitis riparia* × *Vitis rupestris*) and M1 (106/8 [*V. riparia* × (*V. cordifolia* × V. *rupestris*)] × V. *berlandieri* cv. Ressequier n.1) rootstock genotypes were obtained from rooted canes and grown in hydroponic culture in controlled conditions, with a relative humidity of ~65–75%, photoperiod of 16 h light/8 h dark, PAR of 200 mmol m^−1^s^−1^ and temperature ranging from 25°C day/21°C night. Hydroponic cultures were set up into plastic light-reflecting containers, to avoid formation of algae within the nutritive solution. Standard solution, obtained from a modification of the Hoagland solution, was composed of 2 mM Ca(NO_3_)_2_, 0.75 mM K_2_SO_4_, 0.5 mM KH_2_PO_4_, 0.65 mM MgSO_4_, 0.1 mM Fe-ethylenediaminetetraacetic acid (EDTA), 10 μM H_3_BO_3_, 0.5 μM CuSO_4_, 0.5 μM ZnSO_4_, 1 μM MnSO_4_, and 0.05 μM (NH_4_)_6_Mo_7_O_24_. The experiments were carried out using six rooted rootstocks per tank (20 L). The solution was aerated bubbling filtered air and was changed every week. Plants were left into a half-nutritive solution for 10 days, in order to allow them to adapt to the growth conditions. Subsequently, stress treatments were imposed according to Donnini et al. (2012). Acclimated plants were transferred into containers containing a modified nutritive standard solution according to the different conditions applied: (i) control (+Fe): 100 μM Fe-EDTA (pH 6 ~ 6.2); (ii) absence of iron (−Fe): 0 μM Fe-EDTA (pH 6 ~ 6.2); (iii) presence of Bicarbonate (FeBic): 100 μM Fe-EDTA, 10 mM NaHCO_3_, and 0.5 g/L CaCO_3_ (pH 8.2 ~ 8.3). The choice to culture plants within a liquid instead of solid-agar culture was taken in order to avoid perturbations during the experimental procedure, such as the physical injury of plants during transferring from normal to stress conditions and the roots carry-over of residual amounts of Fe-containing agarose. The system allowed the bulk transfer of plants with intact roots into Fe-sufficient (+Fe), Fe-deficient (−Fe), and Fe-limiting (FeBic) media within seconds and with minimal mechanical damage. After 10 days of hydroponic culture in the modified solutions, the root apices of FIVE plants *per* condition (+Fe, −Fe, FeBic) were collected and pooled together for biochemical and molecular analyses. Only actively growing root apices were sampled, trying to collect only those ones that were exposed to the entire kinetic of stress and avoiding the new ones generated during the experimental trial. Sampling was performed in three biological replicates consisting of three independent experiments.

### Physiological and biochemical analyses of 101.14 under different growth conditions

Leaves and roots of 101.14 plants grown in the above mentioned conditions were analyzed by mean of Inductively Coupled Plasma-Mass Spectrometry (ICP-MS) to determine their content in macro- and microelements. Sampled tissues were dried and then mineralized in HNO_3_ by using a Microwave Digestion System (Multiwave ECO). In order to reduce Fe apoplast contamination, roots were collected and rinsed with a solution containing 0,5 mM CaSO4, 10 mM of Na EDTA for 30 min and washed with distilled water several times. Ionome content was determined by ICP-MS (Aurora M90 BRUKER). At the shoot level, gas exchanges were measured with CIRAS portable instrument according to Rolli et al. (2015) and chlorophyll content were determined by using DUALEX system according to Cerovic et al. (2012) on three expanded leaves per plants from three different plants for each treatment. Data collected for both ionome profile as well as *in vivo* leaf physiological analyses are from three independent experiments.

### RNA extraction and libraries construction

Total RNA for both mRNA-Seq and RT-PCR was extracted from ~80 mg of frozen root apices sampled 10 days after imposition of the stress using the “Spectrum™Plant total RNA Kit” according to manufacturer's instructions (Sigma®). RNA quality and concentration were determined by both UV spectrophotometry (Nanodrop™, Thermo Scientific™) and gel electrophoresis analyses. NGS analysis was performed in 101.14 plants on two biological replicates per each condition. mRNA was purified from the total RNA using the Dynabeads mRNA Direct kit (Invitrogen pn 610.12). A variable quantity of mRNA ranging from 0.4 to 1.6% with respect to the amount total of RNA was obtained. Samples for ligation sequencing were prepared according to the “SOLiD Whole transcriptome library preparation” protocol (pn 4452437 Rev. B). Before RNase III digestion samples were purified with Purelink RNA micro kit columns (Invitrogen™), digested from 3′ to 10′ depending on the starting amount of mRNA, retro-transcribed, size-selected using Agencourt AMPure XP beads (Beckman Coulter pn A63881) and barcoded during the final amplification. Obtained libraries (2.5 samples per lane) were sequenced using Applied Biosystems, SOLiD 5500 XL, producing paired-end reads of 75 and 35 nucleotides for the forward and reverse sequences, respectively. Reads were aligned to the v1 prediction of grapevine PN40024 reference genome (http://genomes.cribi.unipd.it/grape) using PASS aligner (Campagna et al., 2009). The percentage identity was set to 90% and one gap was allowed whereas the quality filtering parameters were set automatically by PASS. A minimum reads length cut-off of 50 and 30 nt was set for the forward sequences and reverse reads, respectively. The PASS-pair tool of the PASS package was used to perform the pairing between the forward and the reverse reads and to selected only those sequences that were uniquely aligned. Finally htseq-counts program (http://www.huber.embl.de/users/anders/HTSeq/doc/count.html) was used to quantify gene abundance.

### mRNA-seq analyses and bioinformatics

Statistical analyses for discovering differentially expressed genes (DEGs) were performed with DEseq2 R package (http://www.r-project.org/; Love et al., 2014) according to an FDR-corrected *p* < 0.05. Functional and Gene Ontology associations were inferred from an improved version of the VitisNet Gene Annotation Reference file (Grimplet et al., 2012). Graphical representation of genes expression, hierarchical, and k-Means (KPM) clustering were performed using the Multi Experiment Viewer software (MeV; Saeed et al., 2006) using the Pearson correlation coefficient. Venn diagrams and identification of common and specific DEGs were performed using Venn diagram plotter (https://omics.pnl.gov/software/venn-diagram-plotter).

### Real time reverse transcriptase-PCR

For quantitative real-time PCR analyses (qPCRs) cDNA was synthesized starting from 1 μg of total RNA using the “Transcriptor First Strand cDNA Synthesis Kit” (Roche) with the oligo (dT)_20_ primer according to manufacturer's instructions. qPCRs were carried out in three technical replicates for each sample. Gene specific primers were designed using Primer3 Plus software (Untergasser et al., 2007) and are the following ones: *LDOX* (VIT_06s0004g00770), 5′- AAGCGAGGTCATCGGTCCTT-3′ and 5′- TCTTCCCATCGTGTGCCTTC-3′; *FERRITIN1* (VIT_13s0067g01840), 5′- GCAGGTGGAAGCCATTAAAA-3′ and 5′- GCAGCAACAACTCCTCCATT-3′; *NODULIN* (VIT_19s0093g00320), 5′- GGTAAGAGGTGCATGGAGGA-3′ and 5′- TTCCTGTAATCCACCACCAAG-3′; *AHA2* (VIT_11s0052g00620), 5′-GACTCTCCATGGCCTTCAAC-3′ and 5′- ACCCTTCAGCTTGACCACTG-3′; *IRT1* (VIT_10s0042g01100), 5′- CGGAAATCGAAGTTGCAGAT-3′ and 5′- GCTGCCACAAGAGGCTTTAT-3′; *FRO2* (VIT_15s0046g01900), 5′- TCCGCTTTCGGGTGCCCCTT-3′ and 5′- TGGCGCCAAGGCATAGCCAG-3′; *bHLH38/39* (VIT_08s0007g05300), 5′- TTCAGCAAACAGGCTCAGTG-3′ and 5′- TCACTCAACACCTCGCACTC-3′; *POPEYE* (VIT_17s0000g06000), 5′- TGCCTCAAAAAGGAGAATGC-3′ and 5′- GGAGCAGAGTTGAGGTCAGG-3′; *BRUTUS* (VIT_17s0000g03440), 5′- TTGCCTGGGGATGAAGTTAG-3′ and 5′- GACCAACAAAGCATCAAGCA-3′. Melt curve analysis, agarose gel electrophoresis, and DNA sequencing validated the absence of illegitimate cross-amplification of other genes. Real-time PCR were performed using a Sybr green method on a StepOne Plus Real-Time PCR System (Applied Biosystems™) thermal cycler. Each 10 μl PCR reaction contained 0.6 μl of each primer (10 μM), 1 μl of diluted cDNA, 2X Fast Start Sybr green (Applied Biosystems™) and sterile water. The thermal cycling conditions used were 94°C for 10 min followed by 40 cycles of: 95°C for 30 s, 60°C for 30 s, and 72°C for 30 s, followed by a melt cycle with 1°C increments from 55 to 96°C. Real time PCR data processing was performed using the Δ(ΔCT) method. After testing the suitability of ubiquitin, actin and elongation factor for use of reference genes, elongation factor (VIT_12s0035g01120) was selected for normalization of all samples analyzed.

## Results and discussion

### Characterization of 101.14 morphological and physiological parameters under +Fe, −Fe, and FeBic conditions

**Figure 1:**
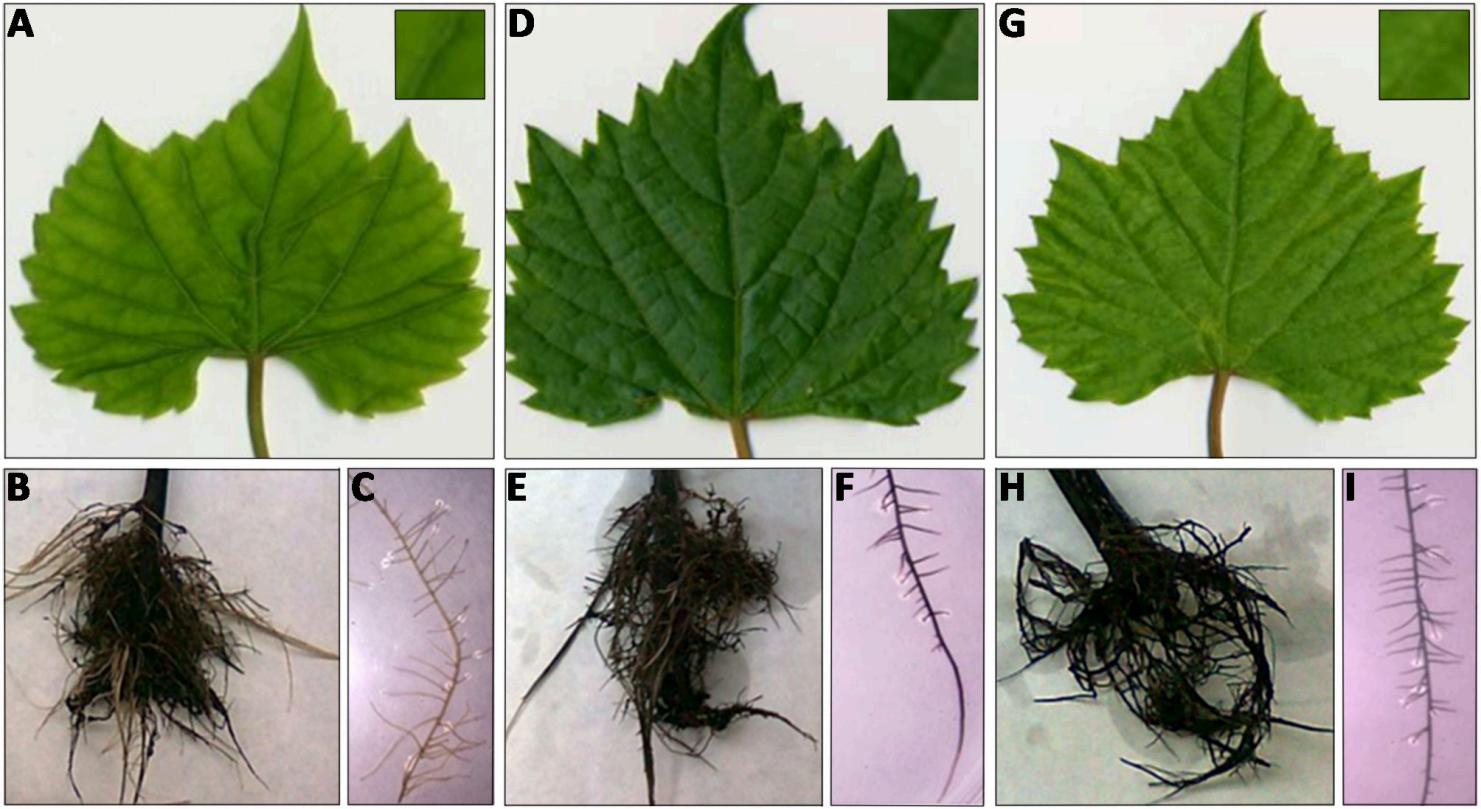
**Shoot and root apparatus of 101.14 plants grown under different Fe-supply conditions**. Leaf detail of 101.14 plants grown upon −Fe **(A)**, +Fe **(D)**, and FeBic conditions **(G)**. Root apparatus and relative detail of −Fe **(B,C)**, +Fe **(E,F,)**, and FeBic **(H,I)**.

**Figure 2:**
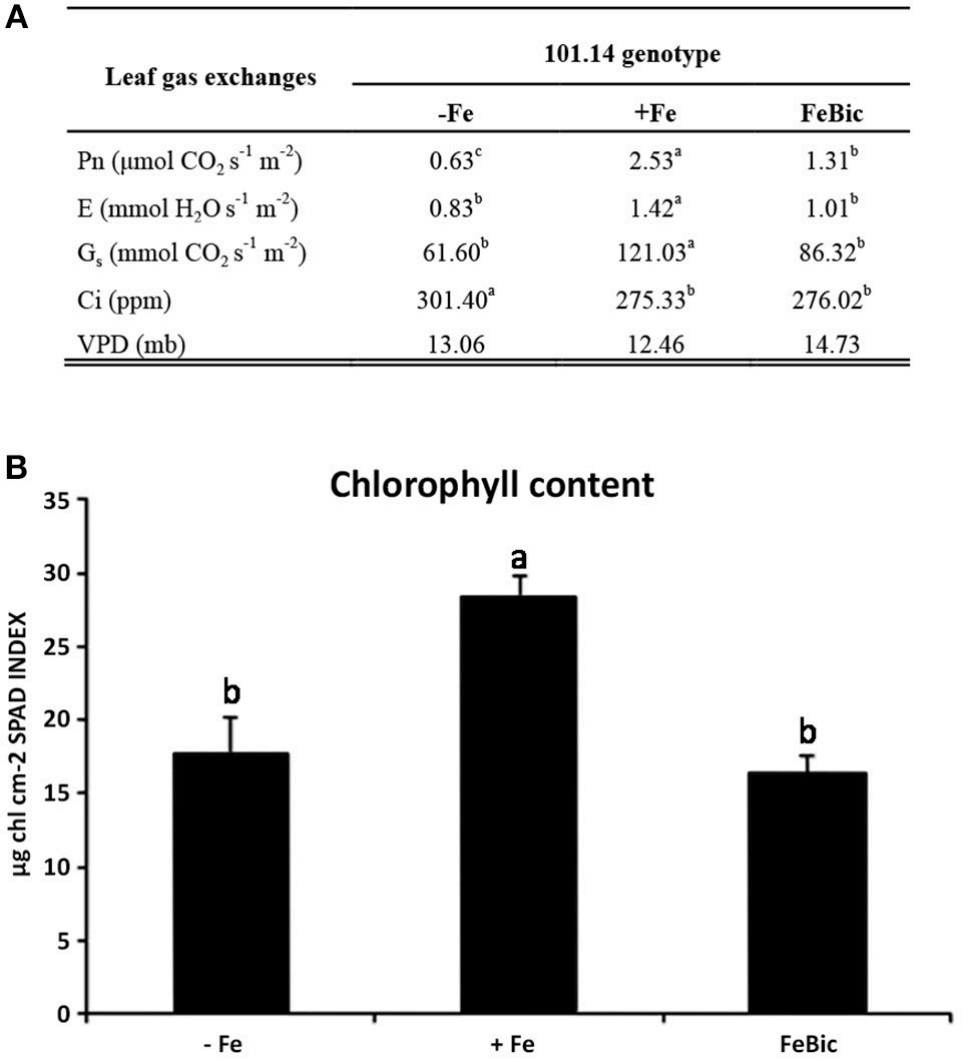
**101.14 physiological parameters upon +Fe, −ig, and FeBic. (A)** Leaf gas exchange parameters were measured by means of portable CIRAS instrument. Values followed by different letters (a, b, and c) are statistically different (test ANOVA, *p* < 0.05). Pn, net photosynthesis; E, evapotranspiration; Gs, Stomatal conductance; Ci, CO2 internal concentration; VPD, Vapor Pressure Deficit. **(B)** Chlorophyll content (SPAD index) upon –Fe, Fe, and FeBic conditions.

After 10 days of culture in both Fe impaired conditions (−Fe and FeBic), both leaves and roots of 101.14 rootstocks showed the typical symptoms of chlorosis compared to control (Figure 1). At the shoot level, the widespread yellowing of leaf internerval tissue was coupled to the decrease of net photosynthesis (*Pn*), stomatal conductance (*G*_*s*_), and transpiration rate (*E*) (Figure 2A), together with a reduction in chlorophyll content determined as chlorophyll meter readings (SPAD index; Figure 2B). Intracellular carbon dioxide concentration (Ci) was significantly increased only in plants grown in −Fe conditions whereas it maintained levels similar to control (+Fe) in plants grown in presence of bicarbonate (Figure 2A). According to previous observations on other plant species, such as tomato (Zamboni et al., 2012), the root apparatus of Fe-deprived 101.14 plants developed more lateral roots (Figure 1).

### Uptake and translocation of iron and major macro- and micronutrients in 101.14

The scarcity or excess of a nutrient such as Fe in the substrate not only determines an alteration of the concentration of that particular element both in root and leaves tissues, but can significantly alter the profile of many other micro- and macronutrients within the plant resulting in important changes in primary and secondary metabolism. In fact, the minerals or ions taken up by plants can be used by the cells as building blocks for the synthesis of organic molecules, or as catalyzers in enzymatic reactions. Moreover, they play important roles as counter-ion in the transport of ionized molecules by the plant, in the osmotic regulation, and in maintaining the electrochemical potential gradient across the cell. Based on this awareness, leaves and roots of 101.14 plants grown in the above mentioned conditions were analyzed by mean of ICP-MS to determine their content in macro- and microelements (Table 1). A first obvious observation, which leapt out analyzing the root micronutrient profile, was the drop of Fe concentration in both −Fe (848.5 μM g^−1^ DW) and FeBic (989.1 μg g^−1^ DW) conditions with respect to control (+Fe, 2104.2 μg g-1 DW). In FeBic treatment, roots still maintained a basal capacity to take up Fe^2+^ with respect to the condition of total Fe-absence showing values slightly higher than in −Fe, but this was not reflected in leaf tissues, which in FeBic conditions showed values even lower than in −Fe (71.2 μg g^−1^ DW; Table 1), suggesting that bicarbonate could impair not only those processes involved in Fe-uptake at the root level but also in its translocation to the shoot. In most of cases all other micro-nutrients, including manganese (Mn), copper (Cu), zinc (Zn), and molybdenum (Mo) showed an opposite behavior with respect to Fe, being accumulated at higher concentration in roots of plants grown in −Fe conditions. Generally, this trend was not observed in roots of those plants cultured in presence of bicarbonate (FeBic), apart from Cu that was the only microelement showing an increased concentration with respect to control. In all other cases the ionome homeostasis was not significantly affected by bicarbonate compared to control, except for Mo, whose concentration was markedly diminished with respect to control (Table 1). We recently demonstrated that Fe and Mo mutually impact their homeostasis in cucumber plant (Vigani et al., 2016). We indeed observed a strong accumulation of Mo in root of Fe-deficient plants. Particularly, we observed Fe accumulation in Mo-deficient mitochondria as well as Mo accumulation in Fe-deficient mitochondria, suggesting that such cellular compartment plays a central role in the Fe and Mo interaction. We demonstrated that the accumulation of Mo in Fe-deficient plant might be related to the activity induction or protein accumulation of cytosolic-localized (such as nitrate reductase) and mitochondrial-localized (mARC) Mo-containing enzymes.

**Table 1:** **Macro- and micronutrient concentrations (μg/g DW) in leaves and roots of 101.14 plants upon −Fe, +Fe, and FeBic**.

***Macro nutrients***	**Leaves**			**Roots**		
	**−Fe**	**+Fe**	**FeBic**	**−Fe**	**+Fe**	**FeBic**
P	62637b	73119a	22883c	6470b	10744a	5421c
Mg	4647b	4736c	3818a	6617b	9741a	6410b
K	34311b	51468c	51544a	15072c	39565a	34457b
Ca	12550a	10583c	11906b	15072c	16356b	33464a
***Micro nutrients***	**Leaves**			**Roots**		
	**−Fe**	**+Fe**	**FeBic**	**−Fe**	**+Fe**	**FeBic**
Mn	63a	76a	39b	71a	39b	34b
Fe	95b	145a	71c	849c	2104a	989b
Cu	13a	8b	14a	231a	39c	137b
Zn	30	25	31	111a	38b	40b
Mo	3c	4b	41a	60a	26b	18c

*Content in macro and microelements determined by mean of ICP-MS in leaves and roots of 101.14 plants grown upon −Fe, +Fe, and FeBic conditions. Values followed by different letters are statistically different (ANOVA, p < 0.05)*.

Moving up the leaf compartment in both the Fe impaired conditions (−Fe and FeBic) Fe showed a decrease in concentration coupled to an increase in Cu and Zn similarly to what observed in roots. The most interesting observations regarded leaves of plants growth under FeBic conditions, with Mn concentration significantly dropping compared to +Fe and −Fe, and, conversely Mo showing a remarkable increase in its concentration with respect to the other two conditions considered.

For what concerns the major macro-nutrients roots showed a reduced accumulation of phosphorus (P) and magnesium (Mg) compared to control, and this behavior was partially maintained also at the leaf level, although with less significant differences for what concerns Mg. Interestingly in leaves there was a huge drop in P concentration under FeBic growing conditions. A similar pattern of nutrients accumulation between the apical and basal organs of 101.14 plants was also observed for potassium (K), whose concentration was reduced only in −Fe conditions in both organs. Finally, of particular relevance was the increase in calcium (Ca) uptake at the root level. This was particularly evident in plants grown in presence of bicarbonate and was only partially correlated with measures obtained in leaves.

### mRNA-seq analyses of 101.14 rootstock genotype subjected to iron-deficient and iron-limited conditions

**Figure 3:**
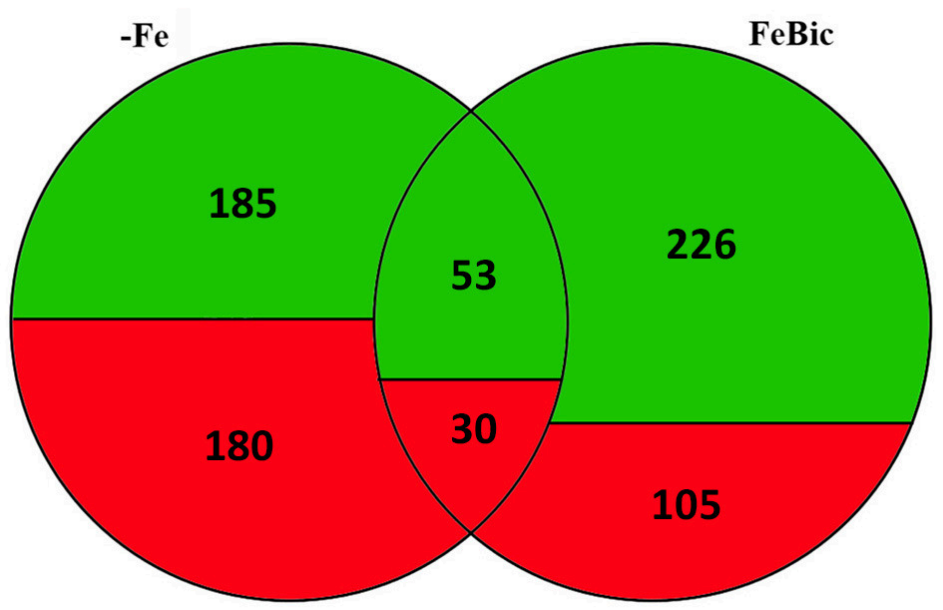
**Venn diagram illustrating Differentially Expressed Genes in grapevine roots in −Fe and FeBic conditions**. Numbers of differentially regulated genes upon −Fe, FeBic or upon both −Fe and FeBic conditions are indicated, together with the relative contribute of up- (red) and down- (green) regulated genes to each subgroup (Fe-specific, common and FeBic-specific).

The whole transcriptomes of root apices collected from 101.14 plants hydroponically grown for 10 days in +Fe, −Fe, and FeBic were sequenced by mean of NGS technology using the SOLiD 5500xl platform. Recently, the genome resequencing of 101.14 indicated that, on average, there is a frequency of approximately one nucleotide variant (SNP) every 200 bases with respect to the PN40024 reference genome (Jaillon et al., 2007; Vitulo et al., 2014; Corso et al., 2015), suggesting the latter represents a suitable reference for read mapping. Thus, all mRNA-Seq reads that passed the quality control test (filtered based on read length after low-quality bases trimming) were mapped on the PN40024 12X v1 grape reference genome (http://genomes.cribi.unipd.it/grape/), producing, on average, ~21 million unique mapping reads per sample (ranging from 18 to 24 million reads depending on the sample). The Principal Component Analysis (PCA) plot (Supplementary Image 1) illustrating the correlation between biological replicates based on mRNA-Seq row filtered reads, showed a good correlation, although those replicates obtained from plants grown under −Fe appeared to be less closely related with respect to those collected from plants grown in +Fe and FeBic. Differentially expressed genes (DEGs) between stressed (−Fe and FeBic) and unstressed (+Fe) conditions are listed in Supplementary Data Sheets 1, 2. As a first general observation, the −Fe and FeBic exposure affected the expression of a similar number of genes (365 DEGs against 331 DEGs, respectively). Eighty-three DE genes (13.5%) were common to both stress treatments, 282 DEGs were specifically regulated in root apices grown in −Fe and 248 in plants grown in presence of bicarbonate (Figure 3; Supplementary Data Sheet 3). In −Fe conditions there was a substantial balance between up and down regulated genes (180 up-regulated and 185 down regulated genes) whereas in FeBic conditions the majority of DE genes were down regulated (Figure 3). A similar trend was observed also for those DE genes that were common between –Fe and FeBic, with the majority of them being down-regulated with respect to the control (Figure 3; Supplementary Data Sheet 3). These results are in agreement with other studies (Thimm et al., 2001; Buckhout et al., 2009; Schmidt and Buckhout, 2011; Zamboni et al., 2012) showing that plant transcriptional response to Fe shortage is based on the modulation of a relative small set of genes compared to other stresses such as water deficit or high salinity (Corso et al., 2015).

### A core set of genes is differentially expressed in both −Fe and FeBic conditions and can be related to the *Arabidopsis* ferrome

**Figure 4:**
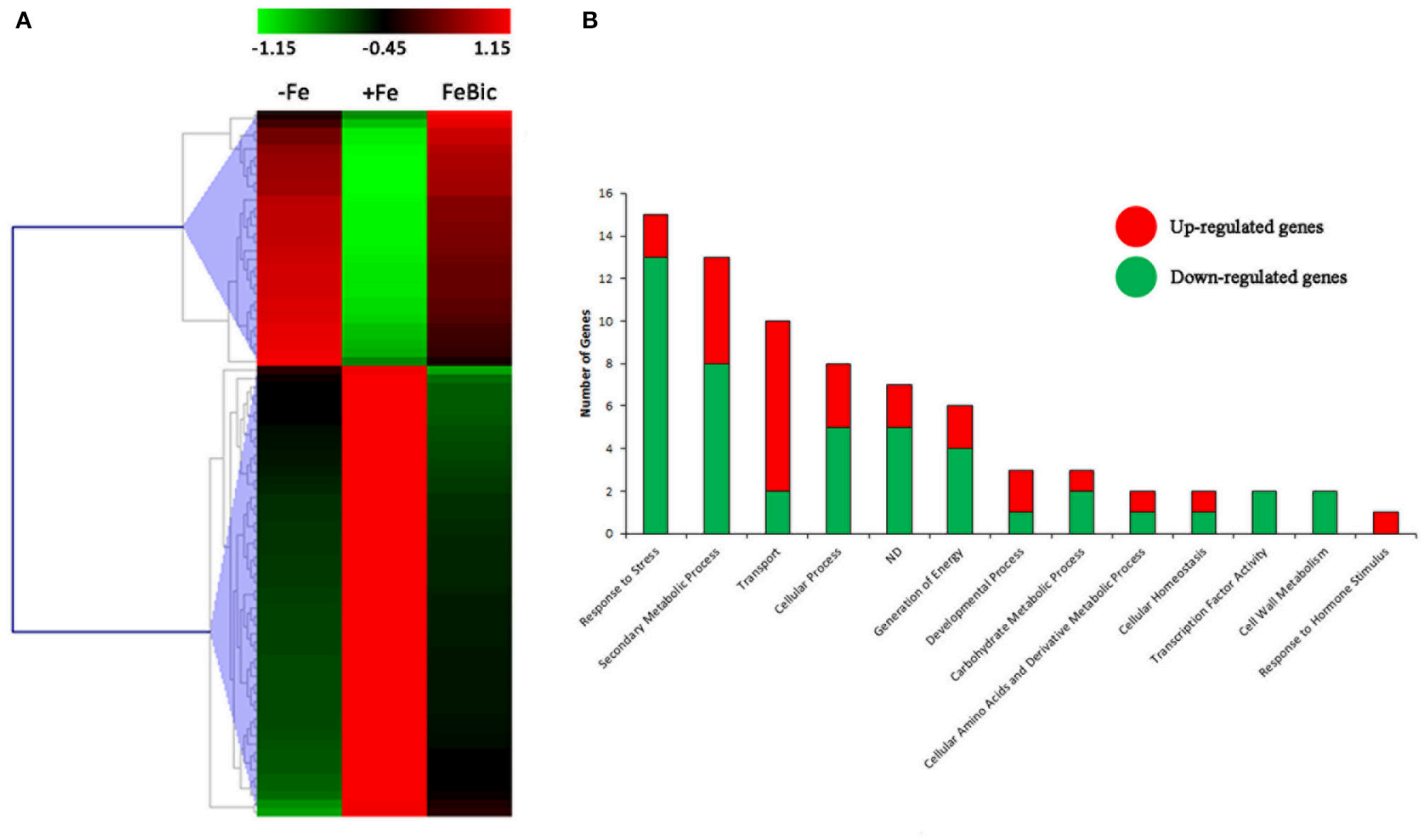
**Heat-map of −ig and FeBic common DEGs and their Gene ontology classification. (A)** The mean unique-read values of DE genes identified in both −Fe and FeBic were graphically represented by mean of MeV software. Hierarchical clustering was performed using MeV HCL tool with the Pearson correlation coefficient. Up-regulated genes are indicated in red whereas down-regulated genes are colored in green. **(B)** Main gene ontology categories related to common DEGs according to VitisNet gene prediction update (Grimplet et al., 2012). For each category the contribute of up- (red) and down- (green) regulated genes is reported.

The majority of DEGs in common between −Fe and FeBic showed the same behavior in both conditions compared to +Fe condition (Figure 4A). Nevertheless, the response observed in complete absence of Fe appeared to be more drastic, especially for stress-induced DE genes, which reached fold-changes (FC) often higher than FeBic (Supplementary Data Sheet 3). Figure 4B shows a manually curated distribution of DE genes in Gene Ontology (GO) categories. Several categories, such as those related to “Response to stress,” “Secondary metabolic process,” and “Transport” were clearly over-represented, with 15 (18%), 13 (16%), and 10 (12%) related-genes, respectively. Whereas the majority of genes belonging to the “Response to stress” and “Secondary metabolic process” were down-regulated (13 genes out of 15 in the “Response to stress” category and 8 genes out of 13 in the “Secondary metabolic process” one) in stressed roots compared to the control ones, the majority of genes belonging to the “Transport” category were induced (Figure 4B). Among them was VIT_10s0042g01100, encoding for *IRT1* (*IRON REGULATED TRANSPORTER 1*), the main transporter involved in Fe^2+^ uptake in Strategy I plants (Lin et al., 2009). Together with *IRT1*-like transcript we identified other genes encoding for transporters such as VIT_00s0259g00140, VIT_00s0259g00120, and VIT_01s0026g01490, all annotated as oligopeptide transporters (OPTs), plasma membrane-embedded proteins, which are involved in the uptake of substrates from the apoplasm and the external environment and in their loading in the vascular system. In particular, VIT_00s0259g00120 is the orthologous of *AtOPT3*, which was demonstrated to transport transition ions *in vitro* and to be involved in phloem in Fe-loading, Fe recirculation from the xylem to the phloem, shoot-to-root Fe signaling, and Fe redistribution from mature to developing tissues (Stacey et al., 2006; Lubkowitz, 2011). VIT_05s0049g00930 was another transcript encoding for a transporter induced in both −Fe and FeBic conditions. In this case the deduced protein is an inorganic phosphate transporter whose role in iron-deficiency has not been described before to our knowledge. Not surprisingly all up-regulated genes encoding for transporters can be associated to related genes in the so called *Arabidopsis*” ferrome,” a core list of 95 transcripts which was identified by Schmidt and Buckhout (2011) and that robustly reflects the transcriptional response of *Arabidopsis* under Fe-deficient conditions. Together with them, many other genes belonging to other ontology categories were related to the ferrome. Among them are genes encoding for two Germin-like proteins (VIT_14s0060g00120 and VIT_14s0128g00650), a purple acid phosphatase (VIT_08s0007g05970), a zinc-finger protein (VIT_09s0054g01740) a cytochrome p450 gene (VIT_13s0067g00110), and most interesting, a bHLH transcription factor (TF), which is orthologous to the Arabidopsis *bHLH38*. Phylogenetic analyses (data not shown) on the amino acidic sequences of predicted bHLH members of the PN40024 genome (Jaillon et al., 2007) and *Arabidopsis* showed that there are three grapevine prediction which cluster with AtbHLH38 (AT3G56970) and AtbHLH39 (At3G56980): namely VIT_08s0007g05300, VIT_13s0073g00400, and VIT_13s0047g00450. Only VIT_08s0007g05300 was induced in both −Fe and FeBic. The other two were listed exclusively in −Fe specifically induced DE genes. In *Arabidopsis* bHLH38/39, together with other members of the bHLH family such as bHLH100 and bHLH101, act together with the binding partner FIT (FER-like Iron Deficiency Induced; Bauer et al., 2007), making heterodimers and activating transcription of downstream iron-related genes such as *IRT1* and *FRO* and thus inducing expression of the Strategy I iron-uptake machinery. Interestingly *FIT* orthologous was not listed on DE genes neither in −Fe and FeBic, being scarcely induced under stress (FC = 1.3 in –Fe and 1.2 in FeBic). A possible explanation of this observation is the fact that *FIT* expression is mainly controlled post-transcriptionally as already demonstrated for its orthologous gene in tomato (Brumbarova and Bauer, 2005).

Amongst those predictions associated to the *Arabidopsis* ferrome were also transcripts undergoing down-regulation, such as those encoding for ferritin 1 (VIT_13s0067g01840) and vacuolar iron transporters-like 5 and 2 (VIT) (VIT_12s0055g00860, VIT_18s0076g00320) proteins. Ferritins are a class of ubiquitous proteins, generally located in chloroplast and mitochondria, which are involved in iron storage and chelation (Petit et al., 2001) and prevent the generation of ROS due to Fe oxidation (Ravet et al., 2009). In the light of this, their down-regulation is most likely due to the lower content of Fe in the −Fe and FeBic samples. VIT (or nodulins-like) are a group of proteins whose function is still object of debate, although recently they have been described to be putatively involved in Fe transport and storage under metal cation sufficiency (Gollhofer et al., 2011). The down-regulation of ferritin transcripts is in line with previous observations in roots of Fe-depleted *Arabidopsis* plants (Thimm et al., 2001; Buckhout et al., 2009; Yang et al., 2010; Stein and Waters, 2011) but also in other species such as tomato (Zamboni et al., 2012). Similarly, Nodulins were described to be down regulated under iron shortage in *Arabidopsis* (Gollhofer et al., 2011), NODULIN-like 1 and 21 in particular (Stein and Waters, 2011). An interesting aspect was the up-regulation of a nodulin-like gene in both −Fe and FeBic conditions. Although this behavior was unexpected, something similar was already observed in tomato roots, where a positive modulation of two transcripts encoding for proteins with a nodulin-like domain was described (Zamboni et al., 2012).

### Genes specifically expressed in absence of iron

**Figure 5:**
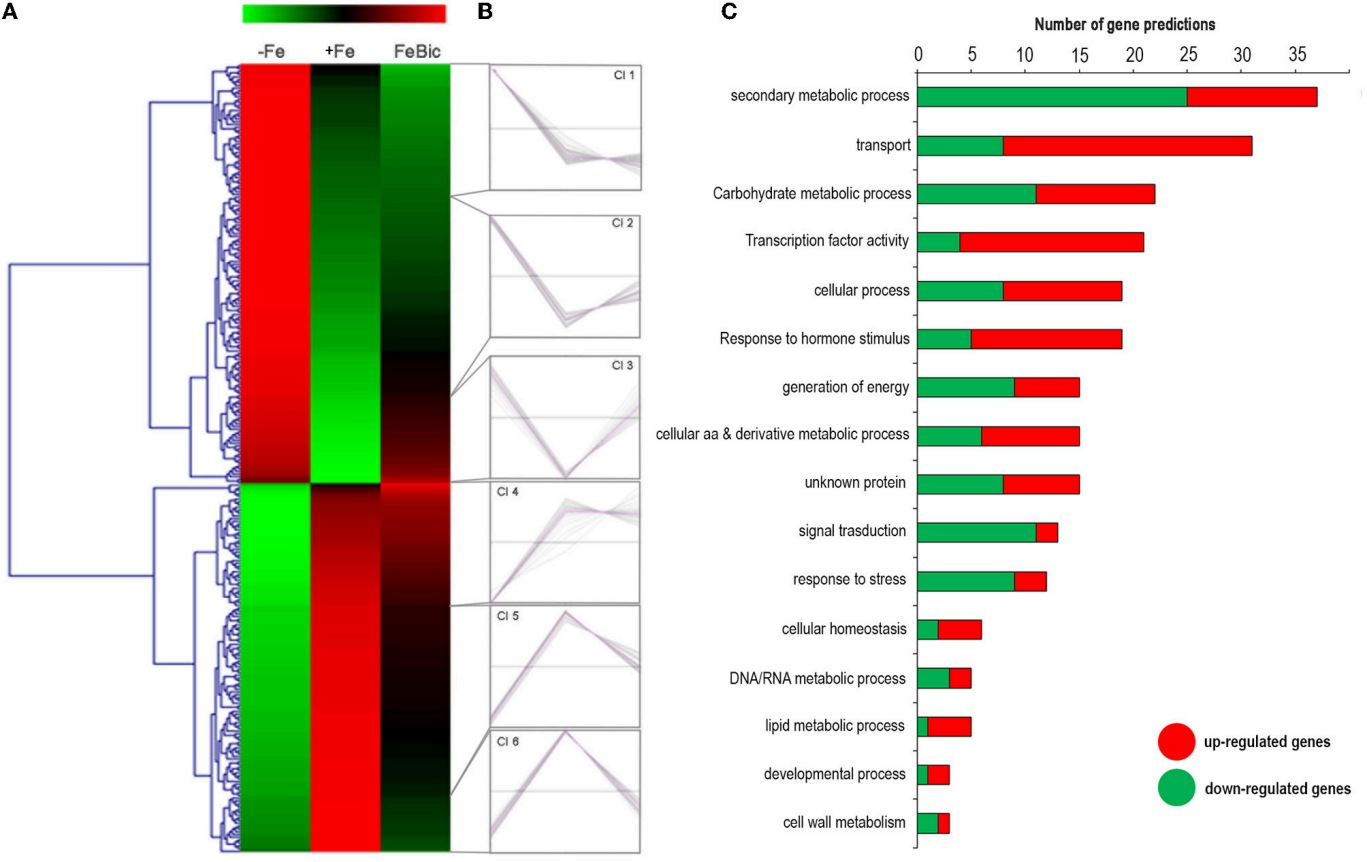
**Heat-map of DE genes specifically regulated in roots grown in absence of iron. (A)** The mean unique-read values of DE genes identified specifically in absence of iron were graphically represented by mean of MeV software. Hierarchical clustering was performed using MeV HCL tool with the Pearson correlation coefficient. Up-regulated genes are indicated in red whereas down-regulated genes are colored in green. **(B)** Expression clusters identified using the k-mean clustering using the Pearson correlation coefficient; **(C)** Main gene ontology categories related to DEGs according to VitisNet gene prediction update (Grimplet et al., 2012). For each category the contribute of up- (red) and -down (green) regulated genes is reported.

DESeq2 R 0 package led to the identification of 282 genes specifically regulated under −Fe. As previously mentioned the behavior of most of −Fe specific DEGs was conserved also in FeBic, although with a weaker response compared to −Fe. This observation is clear looking at the heat map represented in Figure 5A, showing the behavior of −Fe specific DEGs in both conditions analyzed. A k-mean cluster analysis (Figure 5B) showed that among the six clusters that were identified, four were composed of genes showing the same behavior in both conditions (clusters Cl2/Cl3 and Cl5/Cl6 group genes respectively up and down regulated in response to both stresses). More intriguing are clusters Cl1 and Cl4, which contain several genes showing different patterns of expression between −Fe and FeBic. In most cases these genes were induced under −Fe and scarcely modulated or even repressed in FeBic conditions.

Figure 5C illustrates the relative contribute of up- and down-regulated genes to the most-represented gene ontology categories. Of particular interest was the category related to “Secondary metabolic process,” which is the largest in size (up to 38 genes) and encompasses a large proportion of repressed genes in both −Fe and FeBic (Clusters Cl5 and Cl6). Among these, the majority belongs to the grapevine stilbene synthase (*VvSTS*) multigenic family, a large group of structural genes, which have been detected in a limited number of plant species and which are involved in the biosynthesis of resveratrol (three-hydroxy-*trans*-stilbene; Vannozzi et al., 2012; Parage et al., 2012), one of the major grapevine phytoalexins. The extended down-regulation of *VvSTSs* was also coupled to the repression of *VvMYB14* (VIT_07s0005g03340), an R2R3-MYB TF that was demonstrated to actively regulate the expression of *VvSTSs* (Höll et al., 2013) and to the up regulation of several genes belonging to the flavonoid branch, including two flavonoid 3′5′-hydroxylase (*F3*′*H*), a leucoanthocyanidin dioxygenase (*LDOX*) and two UDP-glucose: anthocyanidin 3-O-D-glucosyltransferase (*UFGT*; Supplementary Data Sheet 3). The opposite behavior between genes belonging to the flavonoid and stilbene competing pathways in response to stress was already reported by Vannozzi et al. (2012). In that study, it was observed that both downy mildew (*Plasmopara viticola*) infection and UV-C exposure led to an induction of *VvSTS* genes associated with the repression of three grapevine *VvCHSs* (chalcone synthase), the key genes leading to the flavonoid scaffold.

The fact that 101.14 rootstock appears to drive the flow of carbon trough the synthesis of flavonoid rather than stilbenes under iron deprivation stresses the role for these compounds in the root response to iron deficiency. As a matter of fact, the production of phenolics as root exudates is a well-documented response of strategy I plants to Fe deficiency and include a wide range of molecules, including organic acids, phenolics, flavins, and flavonoids, among others (Schmidt, 1999; Dakora and Phillips, 2002; Cesco et al., 2010; Abadía et al., 2011; Rodríguez-Celma et al., 2013). The composition of these root exudates appears to be genetically determined (Dakora and Phillips, 2002) and it was speculated that excreted phenolic compounds could contribute to the obligatory reduction of iron. Unfortunately, the present study lacks a characterization and quantification of main phenolic compounds secreted by 101.14 roots in the growing media, but this certainly represents an interesting issue to focus on in the future.

As observed for common DEGs, transport represents a GO category particularly enriched in −Fe DEGs, with at least 31 members. As observed for those DE genes encoding for transporters in common with FeBic, also these −Fe specific ones were induced in the majority of cases. Together with VIT_10s0042g01100, we identified VIT_10s0042g01120 and VIT_03s0017g02170, orthologous to *AtIRT1* and *AtZIP12*. Interestingly the latter was amongst those genes showing opposite behavior between –Fe and FeBic, being repressed in −Fe and induced in FeBic. Interestingly, *AtZIP12* was never described as an induced gene in Fe-deprived roots but was identified as a constitutively-expressed gene in the Cd/Zn hyper accumulator *A. halleri* with respect to the non-tolerant *A. thaliana* (Roosens et al., 2008). Many studies indicate that plant metal transporters have wide substrate specificity with limited ion-selectivity and probably the effective combinatorial control of metal transport in a particular deficiency situation must be seen as the result of the expression of a specific mix of transporters with limited selectivity together with high selective transporters such as *IRT1*. Moreover, post-transcriptional regulation should be taken into consideration when analyzing regulation of metal homeostasis, since this regulative process was previously described for *IRT1* in *Arabidopsis* (Connolly et al., 2002). VIT_18s0001g02130 and VIT_07s0129g00620 were other two transcripts showing significant induction under −Fe. They encode for proteins belonging to a different family of membrane-bound metal transporters known as NRAMPs (Natural Resistance-Associated Macrophage Protein; Forbes and Gros, 2001). In *Arabidopsis* AtNRAMP1, AtNRAMP3, and AtNRAMP4 encode functional plant metal transporters and are induced under low iron supply (Thomine et al., 2000). Although the specific role of these transporters in Fe homeostasis remains unclear, it appears likely that they localize to an intracellular compartment, probably to the plastids, as already confirmed for NRAMP1 by Curie et al. (2000). The induction of many aspecific ion transporters under iron shortage could explain the increase in concentration of other micronutrients that we observed thorough ICP-MS (Table 1).

Finally, among the other key genes composing the *Arabidopsis* ferrome we also identified VIT_17s0000g03440, the orthologous of *BRUTUS* (*BTS*; AT3G18290), which encodes a protein with a putative E3 ubiquitin-protein ligase domain and 6 haemerythrin cation binding motifs, which could potentially bind Fe. This protein, BTS, was shown to be co-regulated with PYE (POPEYE; Long et al., 2010), a newly identified TF which plays a critical role in Fe homeostasis and, although its mode of action has not been uncovered yet, it seems to act as a repressor of the iron deficiency stress response since roots of *bts* mutant plants were found to have a higher acidification response when compared to the wild-type and were less susceptible to Fe starvation. Interestingly we were not able to identify *POPEYE* among the DE genes.

### Genes specifically expressed in plants grown in presence of bicarbonate

**Figure 6:**
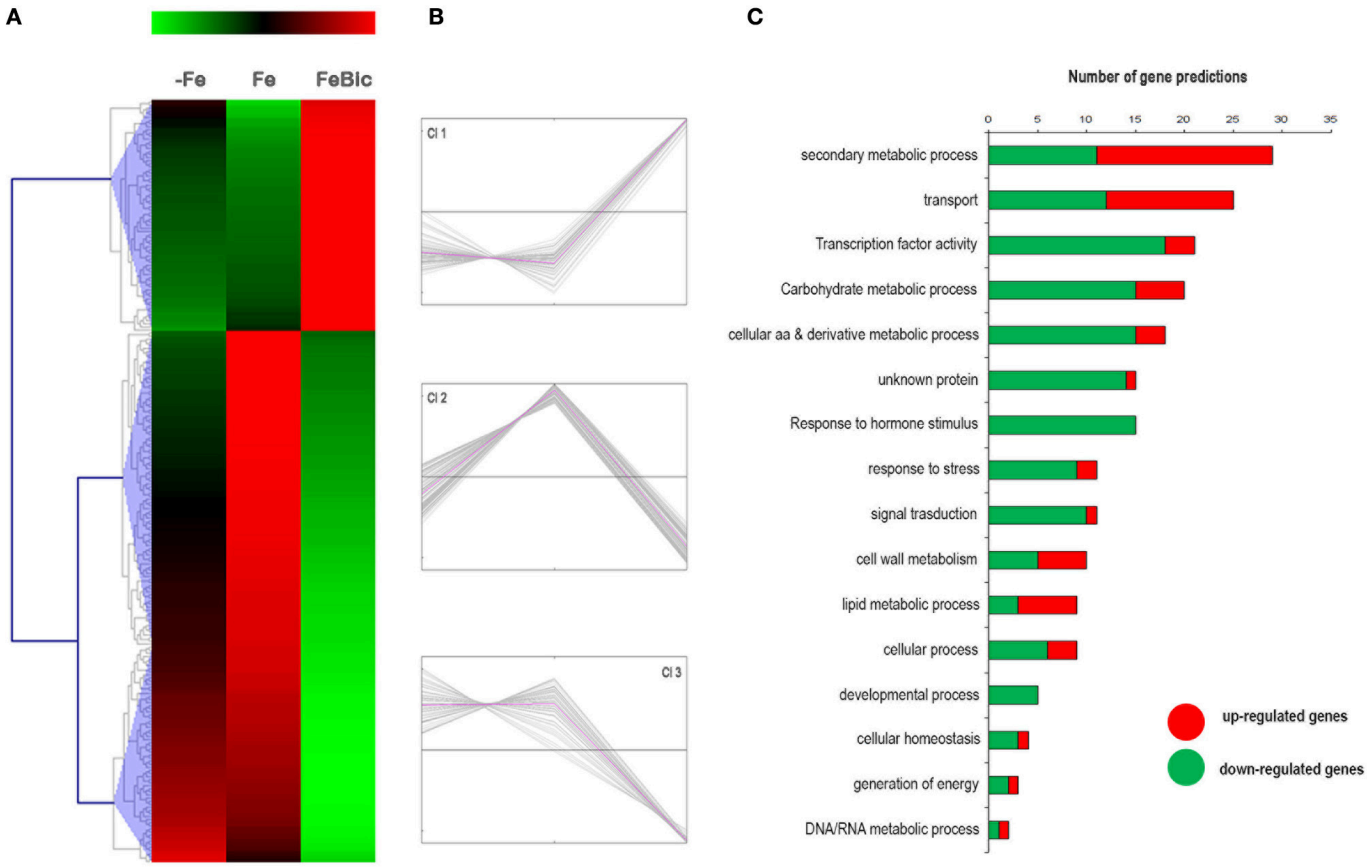
**Heat-map of DE genes specifically regulated in rots grown in presence of bicarbonate. (A)** The mean unique-read values of DE genes identified specifically in presence of bicarbonate were graphically represented by mean of MeV software. Hierarchical clustering was performed using MeV HCL tool using the Pearson correlation coefficient. Up-regulated genes are indicated in red whereas down-regulated genes are colored in green. **(B)** Expression clusters identified using the k-mean clustering based on Pearson correlation coefficient **(C)** Main gene ontology categories related to DEGs according to VitisNet gene prediction update (Grimplet et al., 2012). For each category the contribute of up- (red) and down- (green) regulated genes is reported.

Differing from what observed for Fe-specific DEGs, most of those genes differentially regulated in presence of bicarbonate did not show the same behavior under −Fe, confirming the hypothesis that the presence of bicarbonate leads to a specific response in root apices and should not be simply considered a “less severe” form of iron deprivation. Figures 6A,B clearly show that there are only a few genes, corresponding to those belonging to cluster Cl 2, which are repressed in both stress conditions (anyway the repression observed in −Fe is relatively low compared to FeBic). The remaining DE genes are divided in cluster Cl 1, composed of transcript induced only in FeBic conditions and cluster Cl 3, encompassing genes which are down-regulated only in FeBic. This observation deserves a thought, since many studies on plant response to iron deficiency are based on experiments aimed at mimicking the field conditions by subtracting iron from the growing media. Based on our observations the roots response to a total absence of Fe from the nutritive solution is only partially overlapping with the response observed in presence of Fe plus bicarbonate, and considering the latter condition is more similar to what happens in the field, we believe future research should be oriented at unrevealing the biologic processes involved in the plant response to this particular condition rather than to a complete Fe deprivation. As observed for common and −Fe specific DEGs, also in this case the most represented GO categories were related to “Secondary metabolic Processes,” “Transport” and “transcription factor activity (Figure 6C).”

Among the secondary metabolic processes, several genes encoding for enzyme related to the coumarins synthesis were down regulated (i.e., ferulate 5-hydroxylase; Cinnamoyl alcohol dehydrogenase). As recently demonstrate by several authors, coumarins represent important compound that plants root released into rhizosphere to implement Fe solubility and availability. However, such effect of coumarins has been demonstrated only in Arabidopsis plants, while other plants, such as *Medicago trucantula* are able to release out of the root mainly flavins instead of coumarins (Rodríguez-Celma et al., 2013). Such evidence suggested that the root exudates profiles of Fe-deficient plants might be species-specific. Our result might suggest that response to low Fe availability in *Vitis* spp. might not involve coumarins. Indeed, some other genes involved in the flavonoid biosynthetic pathways (VIT_00s0555g00020, F3′5′H; VIT_07s0005g03150, F3H-like) were up regulated under +FeBic suggesting that other compounds such as myricetin might be likely synthetized in +FeBic-treated plants. Other than secondary metabolites, root exudates released by Fe-deficient plants might involve also carboxylate compounds, such as citrate. It is known that carboxylates are actively released by plants through secondary active transport mediated by protein carriers at the plasma membrane, and the exudation of citrate most probably relies on the activity of MATE transporters (Magalhaes et al., 2007; Valentinuzzi et al., 2015). Interestingly, in the Transport GO category we observed a down regulation of three genes encoding for MATE transporters (Supplementary Data Sheet 3) in +FeBic treated plants, suggesting that probably in 101.14, also the exudation of citrate should not be induced under low Fe availability caused by the presence of bicarbonate. Nevertheless, it's worth to note that MATE transporters are not only involved in the citrate transport but they have a vast range of roles in the mobilization of many other phenolics.

The decrease of both coumarins-synthesis related genes and MATE transporter are more pronounced in +FeBic than in −Fe, suggesting that the exudation of root compounds likely changes when bicarbonate is present in the external medium. Interestingly, with respect to the −Fe, +FeBic treatment strongly induce the expression of some genes related to phosphate homeostasis: VTI_11s0016g05330 (*SPX2*), VIT_07s0005g03290 (Inorganic phosphate transporter 1–4), and VIT_01s0011g06290 (Purple acid phosphatase 3). It is known that calcareous soil limit P availability for plant, therefore 101.14 plant growing under +FeBic by facing also to P deficiency might induce some P-responsive genes.

### Comparison between 101.14 and M1 a new experimental tolerant genotype

**Figure 7:**
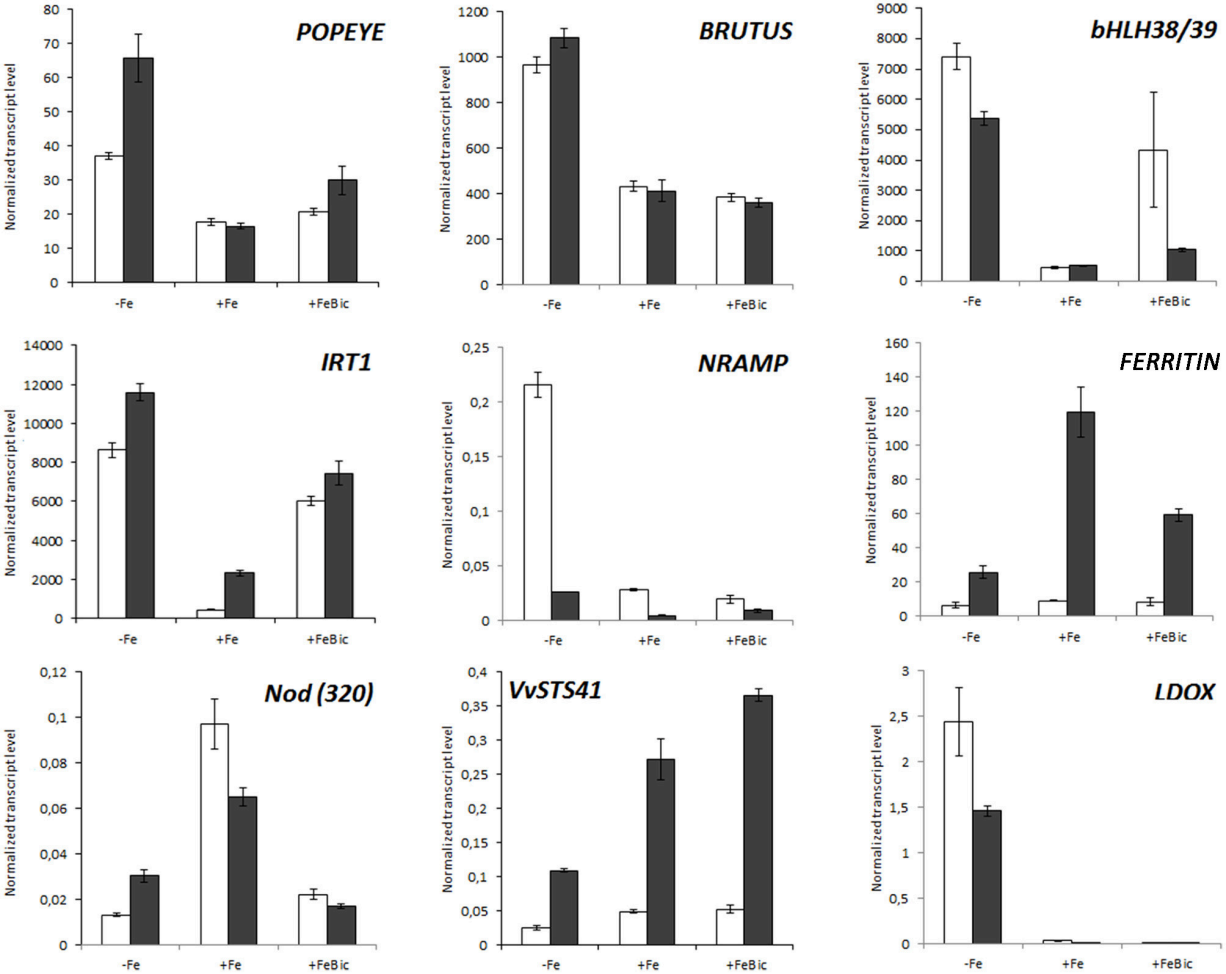
**Real-time RT-PCR validation of a set of genes differentially expressed in mRNA-Seq analyses in 101.14 and in an experimental tolerant rootstock (M1)**. A subset of DE genes was screened by qPCR upon −Fe, +Fe, and FeBic conditions both in 101.14 M1 genotypes. Transcript were normalized to the expression of elongation factor (*EF1*) and plotted as normalized transcript level. Bars indicate standard error (SE) in three technical replicates. Empty columns represent 101.14 genotype whereas dark gray bars represent M1. The gene considered in qPCR analyses were *LDOX* (VIT_06s0004g00770), *FERRITIN* (VIT_13s0067g01840), *NODULIN* (VIT_19s0093g00320), *AHA2* (VIT_11s0052g00620, *IRT1* (VIT_10s0042g01100), *FRO2* (VIT_15s0046g01900), *bHLH38/39* (VIT_08s0007g05300), *POPEYE* (VIT_17s0000g06000), *BRUTUS* (VIT_17s0000g03440).

The expression of set of selected genes, which were differentially expressed in our mRNA-Seq analyses, was checked by quantitative RT-PCR (qPCR) in a third biological replicate constituted by and experiment performed independently from those used for NGS whole transcriptome analyses. We also included the expression of genes which surprisingly were not listed within the DE genes but that are known to be part of the Arabidopsis ferrome and to be involved in the Strategy I mechanism of iron uptake such as *PYE (POPEYE), AHA2* and *FRO2*. In this new experimental trial, stresses were imposed not only to 101.14 plants, but also to another rootstock genotype, named M1. This genotype is characterized by a high degree of tolerance to iron chlorosis, high performances to grafting, reduced vigor and medium resistance to salinity and was recently registered to the National Register of grapevine varieties (http://catalogoviti.politicheagricole.it/crediti.php). Differently from 101.14, the physiological data obtained in M1 under Fe-limited availability pointed out that *Pn, GS* and *E* are similar to control (Supplementary Image 2) without evident symptoms of chlorosis. Figure 7 shows the normalized transcript level of nine iron-related genes including *IRT1*, a *FERRITIN1*-like gene, *bHLH38*/39, a *NODULIN*-like gene, and *VvSTS41* in both the genotypes. For what concerns 101.14, results confirmed what observed in mRNA-Seq analyses, but also highlighted several differences between the two genotypes under study. For example, in both genotypes the *IRT1* transcript was strongly induced in both −Fe and FeBic conditions, but the expression in +Fe condition in M1 genotype was almost six times higher than that observed in 101.14. Also the induction registered under stress was stronger than 101.14. This observation, together with the stronger expression *FERRITIN*-like gene in M1 genotype with respect to 101.14, could be related to a higher capacity of M1 to uptake and to store iron already in unstressed condition. All these results are also supported by the biochemical analysis that pointed out a higher accumulation of Fe under FeBic conditions in M1 than in 101.14. Looking at the expression of genes encoding for transcriptional regulators such as *BRUTUS* and *POPEYE*, both the genotypes showed similar behaviors, although M1 showed a stronger induction of *PYE* in −Fe. Also stilbene synthase and leucoanthocyanidin oxidase genes (*VvSTS41* & *LDOX*) confirmed what observed in the mRNA-Seq data for what concerns 101.14 and highlighted some differences between the two rootstock genotypes under study, at least in terms of entity of the response, although the patterns of expression were comparable. In fact, in both genotypes *VvSTS41* was down regulated under −Fe and was coupled to a remarkable up-regulation of *LDOX* confirming that there must be some involvement of the flavonoid pathway in the plant adaptation to iron-impaired conditions.

**Figure 8:**
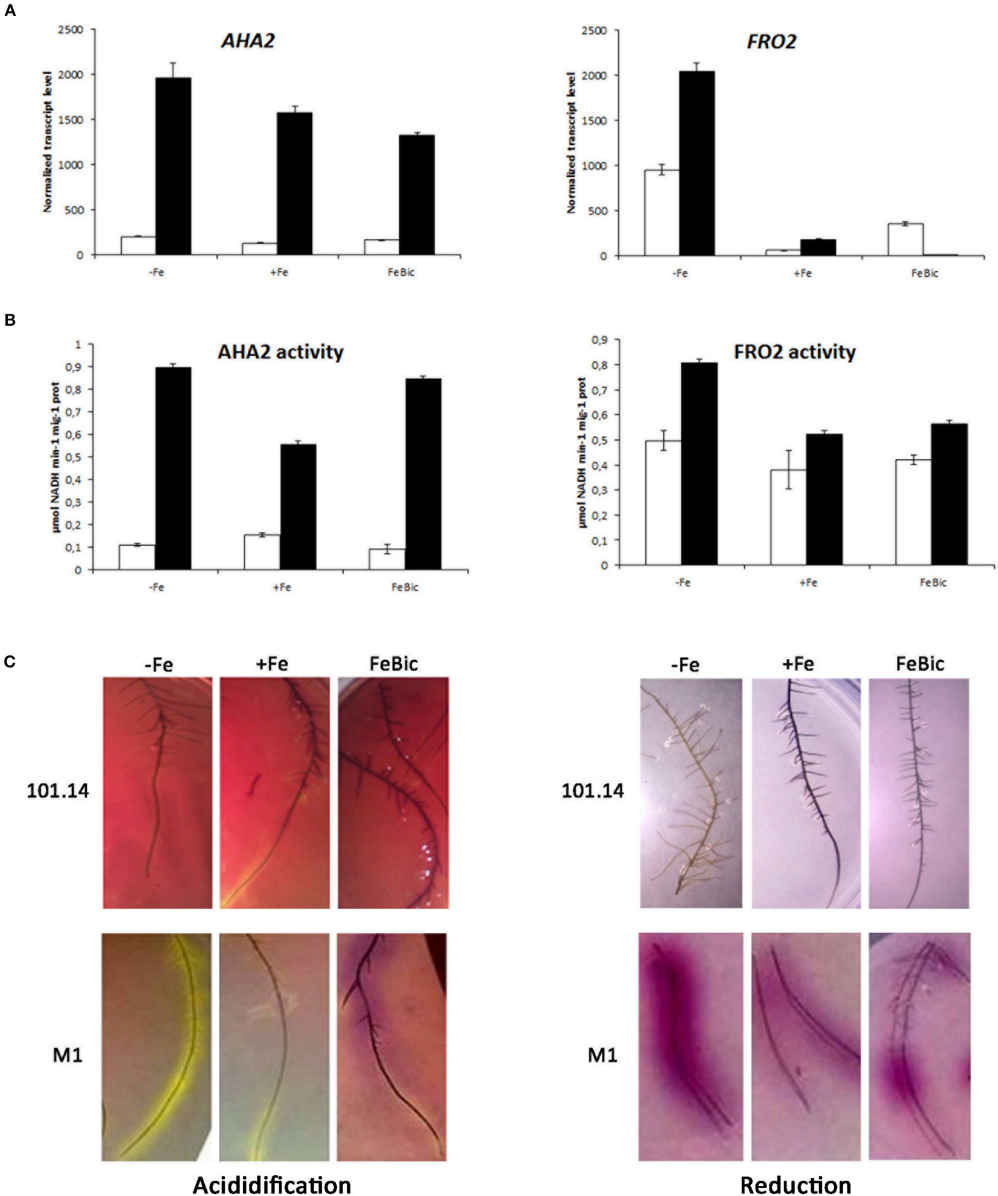
**Gene expression and enzymatic activity of AHA2 and FRO2 in 101.14 and M1 genotypes upon −Fe, +Fe and FeBic conditions. (A)** Expression analyses of *AHA2* and *FRO2* transcript in both 101.14 and M1 rootstocks; **(B)** measure of the H^+^/ATPase and FeIII-chelate reductase activity; **(C)** evaluation of the root ability to acidify the substrate and reduce iron in agar inclusions.

An aspect of particular interest regards the transcript accumulation of genes encoding for *AHA2* and *FRO2*, two key genes involved in the Strategy I iron uptake mechanism. These genes were considered separately from the other ones analyzed by qPCR since we associated to their expression (Figure 8A) also other biochemical analyses such as the measure of the enzymatic activity (Figure 8B) and the evaluation of the root ability to acidify the substrate and to reduce Fe in agar inclusions (Figure 8C). The first interesting observation is the fact that the expression of *AHA2* in M1 genotype is much higher compared to 101.14 in all conditions considered, suggesting a better ability of this genotype to extrude protons in the apoplast. This observation was confirmed at the level of enzymatic activity, which ranged between 0.6 and 0.9 μmol NADH min^−1^ mg^−1^ prot in M1 (in +Fe and −Fe, respectively) and between 0.1 and 0.15 μmol NADH min^−1^ mg^−1^ prot in 101.14 and by the higher capability of M1 to acidify the rhizosphere. All together these observations suggest that the typical strategy I responses are strongly active in M1 while are weak, more delocalized, and diffused in 101.14.

Something similar happened for *FRO2*, the gene encoding for an iron deficiency-inducible ferric chelate reductase responsible for reduction of iron at the root surface. Also in this case the measure of the enzyme activity and the *in vivo* evaluation measured using BPDS confirmed a higher activity of M1 genotype compared to 101.14. Nevertheless, the correlation between biochemical and molecular data was less clear since the expression of *FRO2* gene showed a different pattern respect to the enzyme activity, with a total absence of transcript in M1 in FeBic. A possible explanation relies on the fact that, although focusing on VIT_15s0046g01900, which was the closest *AtFRO2* orthologous in grapevine, we identified at least other three predictions, which cluster with *AtFRO2* (Atg01580). Thus, the effect observed in the biochemical analyses could be due to the synergic action of all or part of these enzymes although at the transcriptional level they could show different patterns of expression. In other words, the activity observed in FeBic conditions could be due to the action of other grapevine *AtFRO2* orthologs with a higher level of expression than VIT_15s0046g01900.

## Conclusions

Low iron bioavailability is a primary constraint to plant growth in many ecosystems, particularly in calcareous soils commonly found in the Mediterranean basin. Thus, iron chlorosis is one of the most frequent nutritional problems in grapevine production. The present study was performed in order to provide a first general description of the transcriptome profile in root apices of a commonly used rootstock genotype under iron deficiency. Not only we analyzed the transcriptome effect of iron deprivation itself, but we also compared it to a condition of low iron availability determined by high concentration of bicarbonate, a phenomenon quite common in nature, which better mimics what happens in the vineyard. To our knowledge this is the first study based on a whole transcriptome analysis describing the response of a grapevine rootstock to iron deficiency. Thanks to this approach we identified many genes potentially involved in the Fe homeostasis in grapevine, some of which differentially modulated in response to both −Fe and FeBic conditions, some others specific to a particular one, suggesting that the root responses to Fe absence and presence of bicarbonate are only partially overlapping and could induce different mechanisms related to different gene regulatory networks. It is important to consider that FeBic treatment, by mimicking a calcareous-like condition, would affect the availability of other nutrients, such as P. Therefore, the partial overlap of results between –Fe and FeBic treatments provides the specific Fe deficiency-induced responses of grape rootstock.

Many DEGs were orthologs of the so-called Arabidopsis ferrome, confirming the grapevine roots behave similarly to Arabidopsis under iron shortage undertaking a Strategy I mechanism of iron homeostasis and representing candidate targets for more focused functional characterization studies. Our results indicated that, together with genes involved in transport, transcriptional regulation, and response to stress, those one related to secondary metabolism are strongly affected by the stresses, as observed for the biosynthetic pathways related to stilbene biosynthesis, which was strongly impaired by stress, and the flavonoid one, some of whose genes were highly induced. Although the 101.14 rootstock genotype is characterized by a high susceptibility to iron chlorosis, the induction of several genes related to the biosynthesis of phenols under stress highlights, once again, the transversal role of these class of compounds in the plant response and adaptation to a vast range of conditions (Ramakrishna and Ravishankar, 2011). These compounds, whose chemical nature has not been described in this study, could be accumulated in the roots with the scope to increase the solubility of iron and its mobilization from insoluble reservoirs and could represent a determinant of tolerance in genotypes characterized by different level of susceptibility to Fe chlorosis. From an agronomical point of view, one of the most interesting outcomes of our study is the different behavior observed for genes involved in the acidification and Fe-reduction processes between 101.14 and M1, another rootstock genotype showing high tolerance to iron chlorosis. In these two genotypes the capability of modulating the strategy I response at the root level, well-correlate with the manifestation of symptoms at the leaf level, suggesting that the activity of AHA2 and FRO2, possibly in association with the capability to accumulate phenolics and transcripts of their biosynthetic genes, could be candidate parameters to be used as functional markers for the selection of new tolerant genotypes.

## Author contributions

AV and ML conceived the design of this study and participated in its coordination; AV performed the bioinformatic data analysis and wrote the paper; SD planned and conducted most of the lab experiments; MC strongly contributed to the statistical analyses of NGS data and in writing the manuscript; GVi and GZ contributed to the interpretation of results and in writing of manuscript; GVa and NV provided NGS platforms and contributed to preliminary bioinformatic analyses; CB and ML contributed to the writing of the paper and critically revised the manuscript. All authors have read and approved the final manuscript.

## Funding

This study was supported by the AGER “SERRES” project, grant n° 2010-2015.

### Conflict of interest statement

The authors declare that the research was conducted in the absence of any commercial or financial relationships that could be construed as a potential conflict of interest.

## Supplementary material

Supplementary Image 1 **PCA plot of mRNA-Seq unique reads**.

Supplementary Image 2 **M1 genotype physiological data upon −Fe, +Fe, and FeBic conditions**.

Supplementary Data Sheet 1 **List of Differentially Expressed Genes identified in root apices of 101.14 rootstock genotype upon −Fe growing conditions**.

Supplementary Data Sheet 2 **List of Differentially Expressed Genes identified in root apices of 101.14 rootstock genotype upon FeBic growing conditions**.

Supplementary Data Sheet 3 **List of common (−Fe + FeBic), Fe-specific and FeBic-specific Differentially Expressed Genes identified in root apices of 101.14 rootstock genotype upon −Fe and FeBic growing conditions**.
