# Supplementary figures and images for: Transcriptional Characterization of a Widely-Used Grapevine Rootstock Genotype under Different Iron-Limited Conditions
Source: Front Plant Sci. 2017 Jan 5;7:1994. doi: 10.3389/fpls.2016.01994 (PMC5214570; doi:10.3389/fpls.2016.01994)

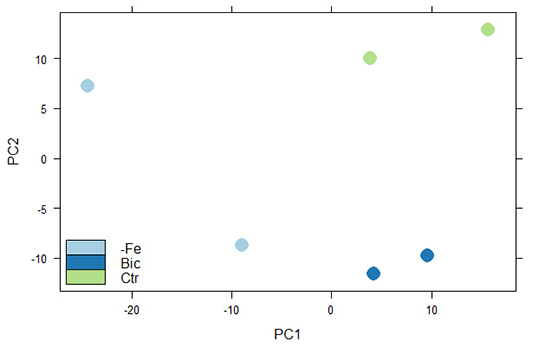

Supplement: Supplementary Image 1 — PCA plot of mRNA-Seq unique reads. [file Image1.TIF]
