# Supplementary material for: Transcriptional Characterization of a Widely-Used Grapevine Rootstock Genotype under Different Iron-Limited Conditions
Source: Front Plant Sci. 2017 Jan 5;7:1994. doi: 10.3389/fpls.2016.01994 (PMC5214570; doi:10.3389/fpls.2016.01994)

Additional file 5 - M1 physiological parameters upon -Fe, +Fe and FeBic.

A) Leaf gas exchange parameters were measured by means of portable CIRAS instrument. Values followed by different letters (a, b, and c) are statistically different (test ANOVA, $p < 0.05$). Pn, net photosynthesis; E, evapotranspiration; Gs, Stomatal conductance; Ci, CO2 internal concentration; VPD, Vapor Pressure Deficit. B) Chlorophyll content (SPAD index) upon –Fe, Fe and FeBic conditions.

**Leaf gas exchanges**	**M1 genotype**		
	**-Fe**	**+Fe**	**FeBic**
Pn (µmol CO_2 s^{-1} m^{-2})	2.46^{b}	5.16^{a}	3.90^{a}
E (mmol H_2O s^{-1} m^{-2})	0.76^{b}	1.16^{a}	1.06^{a}
G_s (mmol CO_2 s^{-1} m^{-2})	51.33^{b}	100.33^{a}	79.34^{a}
Ci (ppm)	264.66^{a}	218.01^{b}	228.05^{b}
VPD (mb)	16.31	12.83	14.72

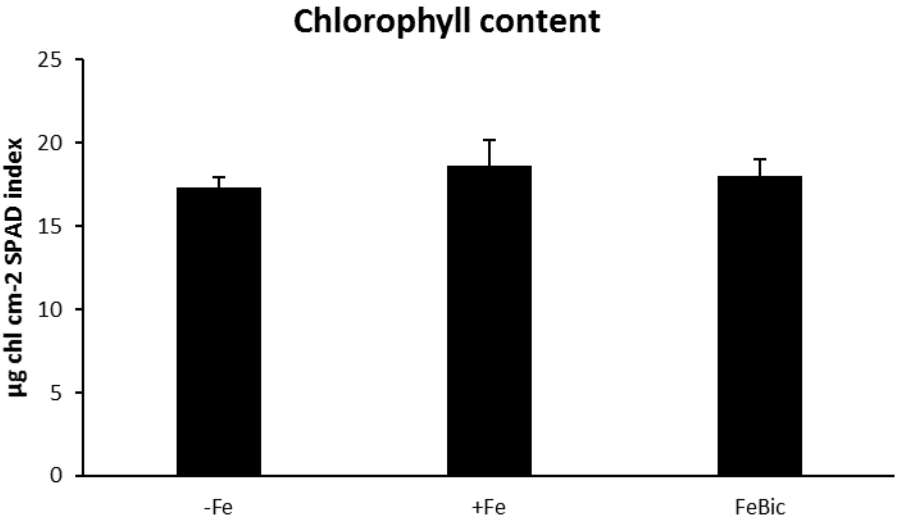

Supplement: Supplementary Image 2 — M1 genotype physiological data upon −Fe, +Fe, and FeBic conditions. [file Image2.PDF]
